# Efectividad de un programa de formación dirigido al abordaje de la violencia de género en profesionales de urgencias y emergencias: Estudio cuasi-experimental

**DOI:** 10.23938/ASSN.1111

**Published:** 2025-04-22

**Authors:** María Gracia Adánez-Martínez, Carlos Pérez-Cánovas, César Leal-Costa, María Belén Conesa Ferrer, José Luis Díaz-Agea, Ismael Jiménez Ruiz

**Affiliations:** 1 Facultad de Medicina Universidad de Murcia Murcia España; 2 Servicio Murciano de Salud Hospital Clínico Universitario Virgen de la Arrixaca El Palmar Murcia España; 3 Instituto Murciano de Investigación Biosanitaria Pascual Parrilla (IMIB) El Palmar Murcia España; 4 Facultad de Enfermería Universidad de Murcia Murcia España

**Keywords:** Violencia de Género, Simulación Clínica, Educación a Distancia, Profesionales de la Salud, Urgencias Médicas, Gender-Based Violence, Clinical Simulation, Distance Education, Health Personnel, Emergency Medical Services

## Abstract

**Fundamento:**

Los sistemas de salud son clave para detectar y abordar la violencia de género. Este estudio evaluó la efectividad de un programa formativo para mejorar los conocimientos, habilidades y actitudes del personal sanitario de urgencias y emergencias frente a esta problemática.

**Metodología:**

Estudio cuasi-experimental con comparación intra-grupo pre-post test. Participaron 115 profesionales de urgencias y emergencias del Servicio Murciano de Salud quienes completaron una formación en línea de 10 horas basado en metodologías activas, con reflexión guiada sobre vídeos didácticos y dramatizados de casos. Se evaluaron los conocimientos, habilidades y actitudes hacia la violencia de género antes y después de la formación utilizando el Cuestionario de Conocimientos, Habilidades y Actitudes sobre Violencia de Género (CCHA-VioGen).

**Resultados:**

Se observaron mejoras significativas en las tres dimensiones evaluadas, con tamaños del efecto medio en conocimiento y habilidad (d=2,07 y d=1,99, respectivamente) y pequeño en actitud (d=0,61). El curso fue valorado positivamente en cuanto a la consolidación del aprendizaje y la aplicabilidad al puesto de trabajo, indicando que el contenido del curso fue percibido como relevante y útil. La necesidad de formación adicional fue baja, sugiriendo que el personal se sintió adecuadamente capacitado con el contenido ofrecido.

**Conclusiones:**

El programa formativo demostró alta efectividad para fortalecer las competencias del personal de urgencias y emergencias en el abordaje de la violencia de género. Ofertar cursos breves y en línea, utilizando recursos innovadores como vídeos-problema dramatizados, se confirma como una estrategia eficaz en contextos con recursos limitados.

## Abstract (en)

**Background:**

Health systems play a key role in the detection and management of gender-based violence. This study aimed to evaluate the effectiveness of a training program designed to enhance the knowledge, skills, and attitudes of emergency healthcare professionals in addressing gender-based violence.

**Methodology:**

A quasi-experimental pre-post study was conducted with 115 emergency health professionals from the Murcian Health Service. Participants completed a 10-hour online training program utilizing active learning methodologies, including guided reflection on didactic and dramatized problem-based videos. The Knowledge, Skills, and Attitudes on Gender-Based Violence Questionnaire (CCHA-VioGen) was administered before and after the training

**Results:**

Statistically significant improvements were observed in all three dimensions. Knowledge and skills showed medium effect sizes (d=2.07 and d=1.99, respectively), while attitude improved with small effect size (d=0.61). Participants rated the course highly in terms of learning consolidation and applicability, indicating that the course content was perceived as relevant and useful. The perceived need for further training was low, suggesting a strong sense of preparedness following the intervention.

**Conclusions:**

The training program is highly effective in enhancing healthcare professionals´ competencies in managing cases of gender-based violence. Short, online courses that incorporate innovative resources such as dramatized problem videos represent a feasible and impactful strategy for professional development, especially in setting with limited resources.

## INTRODUCCIÓN

La violencia de género (VG) es un problema de salud pública transversal a todos los estamentos de la sociedad^1^^,^^2^. La Organización Mundial de la Salud (OMS) estima que una de cada tres mujeres en el mundo han sufrido algún tipo de violencia física y/o sexual en algún momento de su vida^3^. Según datos del Instituto Nacional de Estadística, en 2023 encontramos 368.244 mujeres víctimas de violencia de género en España^4^.

Desde el 1 de enero de 2003 hasta junio de 2024 se han registrado en España 1.258 asesinatos por violencia de género^5^. Pero al número de víctimas y consecuencias mortales se suma el impacto que la violencia de género genera sobre la salud de las mujeres, ya que afecta a todas las esferas de la persona: física, psicológica, sexual, reproductiva y social^3^^,^^6^. Es necesario precisar que la violencia de género afecta tanto a las mujeres que la sufren como a su entorno. Las niñas y adolescentes no escapan a esta circunstancia condicionando su vida desde antes de nacer^7^.

Los sistemas de salud son un entorno privilegiado para la detección y abordaje integral de las mujeres víctimas de violencia de género^7^^-^^9^; siendo a menudo los servicios de urgencias el punto de acceso y detección de estos y otros casos de violencia contra las mujeres^1^. Según el Informe Anual de 2023 de Violencia de Género del Ministerio de Sanidad^10^ en el ámbito sanitario se notificaron 15.301 declaraciones de violencia de género (76% en atención primaria), de los cuales 466 fueron notificadas en la comunidad de Murcia.

A pesar de que la mayoría de las regiones españolas cuentan con directrices y protocolos de actuación sanitaria (Murcia cuenta con un protocolo para la detección de violencia de género en atención primaria^11^) o se basan en el protocolo común para la actuación sanitaria ante la violencia de género publicada por el Ministerio de Sanidad, Servicios Sociales e Igualdad en el 2023^12^, sigue existiendo una gran heterogeneidad en relación a la formación del personal de los servicios sanitarios^13^^,^^14^. Durante el año 2022 se formaron 21.480 profesionales sanitarios en España (7% fueron profesionales de urgencias) mediante 409 acciones formativas (48 en urgencias)^10^. El infradiagnóstico de la violencia de género en el ámbito sanitario es un problema multifactorial^15^^,^^16^ que requiere de un abordaje integral. En ocasiones las víctimas tienen miedo a denunciar el abuso por temor a las repercusiones o el rechazo social, lo que puede llevar a ocultar la violencia en la consulta médica^17^^,^^18^. A esto se suman las barreras culturales y la falta de recursos, entre los que se incluye el tiempo, los recursos institucionales, la capacitación del personal sanitario^8^^,^^19^ o el impacto emocional sobre el personal sanitario que se enfrenta a situaciones de violencia de género^20^.

La atención sanitaria basada en la escucha activa, la ausencia de prejuicios, y la orientación para acceder a otros recursos de ayuda son fundamentales para la detección y cribado^18^. Por tanto, la formación adecuada para identificar y manejar casos de violencia de género es un pilar para reconocer los signos de abuso y responder de manera efectiva^8^. La gestión de los casos de violencia de género no puede depender de las actitudes y prejuicios personales del personal sanitario, por lo que existe la necesidad de garantizar su capacitación para optimizar dicha gestión basada en el respeto a los valores éticos y en competencias relacionales y de aplicación de protocolos y directrices^21^^-^^23^. Así, el Plan Nacional de Sensibilización y Prevención de la Violencia de Género establece como criterios comunes de calidad e indicadores de evaluación, la formación a todo el personal sanitario de los ámbitos de gestión, administración y asistencial que esté implicado directamente en la prestación de servicios de salud a las mujeres con contenidos adecuados a las competencias laborales que requiera el puesto de trabajo^24^.

En este sentido, estudios previos demuestran que la formación en violencia de género aumenta la detección y gestión de casos^21^, puede mejorar los conocimientos y autopercepción de los profesionales sanitarios sobre su preparación para responder a las personas afectadas y, aunque las pruebas que respaldan alguno de estos estudios son débiles e inconsistentes, puede mejorar la identificación y documentación en las historias clínicas de las mujeres^25^.

Las recomendaciones internacionales sobre la necesidad de mejorar los sistemas de urgencias y emergencias en la atención a la violencia de género son cada vez más frecuentes^26^^-^^29^ y numerosos estudios subrayan la importancia de entrenar a los profesionales en la detección y seguimiento de los casos en estos contextos^23^^,^^30^^-^^33^.

Experiencias formativas basadas en metodologías activas de aprendizaje han demostrado ser eficaces para mejorar las estrategias de comunicación y entrevista telefónica para la detección y rastreo de casos COVID-19 y violencia de género^34^^,^^35^. El Servicio Murciano de Salud ha diseñado el *Plan formativo en servicios de urgencias con metodologías activas de aprendizaje* para capacitar a profesionales de salud en urgencias y emergencias. Este plan abarca sensibilización, toma de conciencia emocional, entrenamiento en comunicación y aprendizaje basado en análisis, reflexión y toma de decisiones mediante vídeos problemas inacabados. Las personas participantes se involucran emocionalmente y encuentran soluciones mediante reflexión y técnicas de debriefing^33^^,^^36^. Creemos que estas estrategias, que unen capacitación específica, conocimiento de protocolos de actuación y entrenamiento con metodologías activas de aprendizaje, pueden ayudar a capacitar al personal de los servicios de urgencias y emergencias para la atención de casos de violencia de género.

El objetivo de este estudio fue evaluar la efectividad de un programa de formación con simulación, vídeos didácticos y vídeos-problema dramatizados, en el abordaje de la violencia de género por parte de profesionales del ámbito de las urgencias y emergencias del Servicio Murciano de Salud, mediante la comparación de los conocimientos, habilidades y actitudes antes y después de la capacitación.

## MATERIAL Y MÉTODO

### Diseño

Estudio cuasi-experimental con comparación intragrupo de conocimientos, habilidades y actitudes de profesionales de medicina y enfermería de los servicios de urgencias y emergencias del Servicio Murciano de Salud, antes y después de realizar un programa de formación en violencia de género La recogida de datos se realizó entre enero de 2021 y diciembre de 2023.

Las personas participantes en este estudio se reclutaron mediante un muestreo por conveniencia entre las que realizaron el programa de formación. Se excluyeron aquellas que no firmaron el consentimiento informado y las que habían sufrido violencia de género.

### Programa de formación

Se diseñó un programa de capacitación para abordar la violencia de género con metodologías de aprendizaje activo. La formación se realizó mediante dos sesiones síncronas en línea de 4 horas de duración cada una utilizando herramientas de Zoom. Los contenidos teóricos estaban disponibles en línea en la plataforma de aula virtual antes de cada sesión.

Se combinaron diferentes metodologías activas de aprendizaje práctico: dinámicas de grupo, aprendizaje entre iguales, toma de conciencia emocional sobre las bases del análisis reflexivo de los vídeos de problemas dramatizados. Hubo una interacción constante entre participantes y docentes. Las sesiones síncronas fueron íntegramente prácticas, se realizaron tareas de discusión y reflexión con el foco puesto en el análisis de los vídeos-problema.

Dos personas expertas con más de diez años de experiencia en violencia de género y capacitación en metodologías de aprendizaje activo diseñaron el contenido de los vídeos seleccionando situaciones que abordaban los dilemas más comunes en la toma de decisiones clínicas sobre violencia de género. Diferentes historias de mujeres fueron guionizadas y grabadas en las salas de simulación clínica de la Universidad de Murcia con la participación de actores y actrices profesionales. Se realizaron dos tipos de vídeos; 1) *vídeos didácticos*, para ilustrar el camino o protocolo que se aplica a una víctima de violencia de género cuando busca ayuda en un servicio de urgencias, y la entrevista a la víctima de violencia de género con herramientas de comunicación eficientes. 2) *vídeos-problema* que describen una situación inconclusa y en los que se utilizó una metodología basada en el *debriefing* clínico^37^. Una persona participante asumía el papel de una de las protagonistas del vídeo, identificaba la situación, reflexionaba y buscaba soluciones (planteaba cuestiones a resolver relacionadas con el caso que posteriormente trasladaba a una situación real), realizando las conclusiones del caso. El contenido de los vídeos docentes y problema se puede observar en la Tabla 1.

**Tabla 1:** Vídeos docentes y problema

Video docente	Situación clínica	Objetivos de aprendizaje
*Recorrido / protocolo*	Una mujer llega al servicio de urgencias para ser atendida por VG	- Conocimiento y aplicación práctica del protocolo de VG
*Entrevista con víctima de VG*	Entrevista con víctima de VG	- Aplicar elementos de comunicación eficaz
		- Partes de la entrevista clínica
		- Detección de VG
*Video-problema*	*Situación clínica*	*Objetivos de aprendizaje*
*Las resistencias están entre nosotros*	Debate entre dos profesionales de la salud cuando una de las personas presenta resistencia a atender a una víctima de VG	- Identificar las situaciones en que se pone resistencia y desarrollar argumentos para ayudar a la mujer
*Y si me atreviera*	Sospecha de VG en una mujer que no quiere intervención	- Valoración del riesgo
		- Ofrecer recursos
*El momento de mayor riesgo*	Atención a gestante víctima de maltrato	- Identificar el embarazo como situación de alta vulnerabilidad
		- Conocer protocolos y recursos
*La necesidad de actuar*	Atención a mujer víctima de maltrato con lesión grave	- Conocer el protocolo ante situación de alto riesgo
		- Conocer y ofrecer recursos
*Se trata de una situación sospechosa*	Sospecha de trata de personas	- Identificar la situación
		- Conocer protocolo y recursos
*Deja mi móvil*	Sospecha de maltrato en adolescente	- Sensibilizar sobre el problema de maltrato en adolescentes

VG: violencia de género.

### Instrumentos de medida

Para evaluar el programa de formación en metodología de aprendizaje activo sobre el abordaje de la violencia de género, se utilizó el *Cuestionario de conocimientos, habilidades y actitudes sobre violencia de género* (CCHA-VioGen)^30^, creado y diseñado en 2020 por un panel de ocho expertos con más de diez años de experiencia en violencia de género, y con formación en metodologías de aprendizaje activo, con un índice de validez de contenido (IVC) adecuado tanto para todos los ítems (entre 0,87 y 1) como para el cuestionario total (IVC=0,97); la consistencia interna de la escala total fue adecuada (α=0,79)^30^. Está compuesto por 17 ítems basados en las recomendaciones actuales para abordar a las mujeres que sufren violencia de género y puntuados con una escala de respuesta tipo Likert (1= desacuerdo total a 10= acuerdo total); en la dimensión *actitud* hay tres ítems (1,3,5) redactados negativamente cuya puntuación se invirtió para que en todos los casos una mayor puntuación indique mayor valoración de la variable estudiada. Los ítems se agrupan en tres dimensiones: *conocimiento* (ocho ítems, puntuación entre 8 y 80), *habilidad* (tres ítems, puntuación entre 3 y 30) y *actitud* (seis ítems, puntuación entre 6 y 60 (Anexo I).

- La dimensión *conocimiento* evaluó el conocimiento sobre el protocolo de salud de la violencia de género, sobre las posibles acciones cuando se trata de una víctima de violencia de género, sobre las preguntas de selección para identificar a una posible víctima de violencia de género, los riesgos de una mujer que es víctima de violencia de género, cómo completar y qué hacer con el informe de lesión cuando se trata de una víctima de violencia de género, qué información y apoyo podría ofrecerse a una mujer víctima de violencia de género, las consecuencias de la violencia de género en la salud de la mujer, y una entrevista que se llevará a cabo con una posible víctima de violencia de género.
- La dimensión *habilidad* evaluó aspectos de violencia de género como la capacidad de las participantes para completar el informe de lesiones de las mujeres que padecían violencia de género, hacer las preguntas de cribado de violencia de género y ofrecer los recursos disponibles.
- La dimensión *actitud* evaluó la posición de los profesionales sobre la atención de la violencia de género, el abordaje con los colegas desde el punto de vista de la atención a la salud, si se sentían incómodos y si experimentaban ansiedad al atender a una posible víctima de violencia de género que no quería denunciar al agresor.

### Recopilación de datos

Para la realización de este estudio se obtuvo la aprobación del Comité de Ética del Hospital Clínico Universitario Virgen de la Arrixaca (Código NE-2021-4-HCUVA).

Se diseñó un formulario de recogida de datos con el instrumento de medida CCHA-VioGen a través de la herramienta Google Formularios que se envió a través de correo electrónico para que las personas participantes lo cumplimentaran y enviaran antes de la realización del programa de formación sobre violencia de género con metodología activas de aprendizaje.

A los tres meses de la realización del programa de formación se envió de nuevo el CCHA-VioGen a través del Aula Virtual de la formación, lo que facilitó su distribución y acceso. Además, se agregaron ocho preguntas adicionales elaboradas *ad hoc* para evaluar si las personas participantes poseían mejores herramientas para abordar la violencia de género en los servicios de urgencias y emergencias después de la capacitación. Para ello, se midieron los siguientes aspectos: la consolidación del aprendizaje (tres preguntas), la aplicabilidad al puesto de trabajo (tres preguntas) y la necesidad de formación complementaria o no (dos preguntas) (Anexo II). También se recogieron variables sociodemográficas (sexo: hombre/mujer y edad en años cumplidos) y profesionales (años de experiencia y categoría profesional: medicina/enfermería).

### Análisis de datos

Se realizó un análisis descriptivo de las variables del estudio utilizando media y desviación estándar (DE) para las variables cuantitativas y frecuencias y porcentajes para las variables categóricas. Las puntuaciones pre y post intervención se compararon mediante la prueba t-Student para muestras relacionadas aplicando la corrección de Bonferroni para comparaciones múltiples. Para valorar la magnitud del efecto de la intervención en cada variable se calculó el tamaño del efecto de Cohen (d), interpretándolo según los valores propuestos por Ferguson^38^, donde d=0,41 indica un efecto pequeño, d=1,15 medio y d=2,70 grande. Para analizar los datos se utilizó el software SPSS® v. 25 (*Statistical Package for the Social Sciences*). La significación estadística se fijó en un valor de p <0,05.

## RESULTADOS

La muestra de participantes estuvo compuesta por 115 profesionales sanitarios, lo que representa una tasa de respuesta del 64,61% sobre los 178 inicialmente seleccionados. La media de edad fue 37,55 años (DE=10,95), la mayoría (83,5%) fueron mujeres, el 53,9% era personal enfermero y la media de experiencia profesional fue 8,32 años (con gran heterogeneidad). No se observaron diferencias por sexo (Tabla 2).

**Figura 1:**
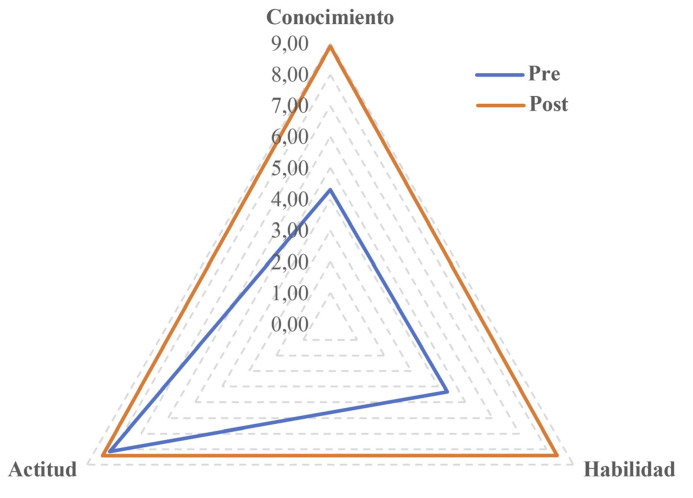
Puntuaciones totales pre y post formación ponderadas sobre 10 obtenidas en el cuestionario CCHA-VioGen. Las puntuaciones fueron significativamente mayores tras la formación recibida (p<0,001).

Tras la acción formativa de abordaje de la violencia de género con metodologías activas de aprendizaje, todos los ítems de las dimensiones *conocimiento* y *habilidad* aumentaron de forma significativa tras la formación, con un tamaño el efecto medio. Sin embargo, las puntuaciones de los ítems en la dimensión *actitud* fueron similares tras la formación, aunque mostraron diferencias estadísticamente significativas (excepto para el ítem 4), con un tamaño del efecto pequeño (Tabla 3, Fig. 1). Los tamaños de efecto fueron medios en las dimensiones *conocimiento* (d=2,07) y *habilidad* (d=1,99), y pequeño en *actitud* (d=0,61).

El análisis desagregado por sexo no mostró diferencias significativas en las puntuaciones pre y post formación para ninguna de las dimensiones del cuestionario CCHA-VioGen (conocimiento, habilidad y actitud).

**Tabla 2:** Características generales de las personas participantes, global y por sexo

Variables	Total	Hombre	Mujer	p
	n=115	n=19 (16,5%)	n=96 (83,5%)	
Edad (años), *M (DE)*				0,881
	37,55 (10,95)	37,89 (11,03)	37,48 (10,99)	
Categoría Profesional, *n (%)*				0,132
Medicina	53 (46,1)	12 (63,2)	41 (42,7)	
Enfermería	62 (53,9)	(36,8)	55 (57,3)	
Experiencia profesional (años), *M (DE)*				0,587
	8,32 (9,41)	9,39 (10,64)	8,10 (9,19)	

M: media; DE: desviación estándar.

**Tabla 3:** Puntuaciones obtenidas en los ítems del Cuestionario de conocimientos, habilidades y actitudes sobre violencia de género (CCHA-VioGen) pre y post acción formativa

Ítems	Formación		Diferencia		
	Pre (n = 115)	Post (n = 115)	pre-post formación		
	M (DE)	M (DE)	M (95% IC)	p	d
*Conocimiento*					
1. Conozco el protocolo sanitario de VG.	4,23	8,77	4,54	<0,001	1,99
	(1,00)	(1,00)	(4,12-4,96)		
2. Conozco cómo actuar al encontrarme en mi servicio con una posible víctima de VG	4,4	8,72	4,32	<0,001	1,85
	(2,16)	(1,01)	(3,89-4,75)		
3. Conozco las preguntas de cribado para identificar a una posible víctima de VG.	4,24	8,86	4,62	<0,001	1,85
	(2,32)	(1,00)	(4,16-5,08)		
4. Conozco los riesgos en la mujer que es víctima de VG.	4,56	9,19	4,63	<0,001	1,88
	(2,19)	(0,84)	(4,18-5,09)		
5. Conozco cómo cumplimentar y qué hacer con el parte de lesiones ante una víctima de VG.	4,09	8,58	4,5	<0,001	1,85
	(2,21)	(1,15)	(4,05-4,94)		
6. Conozco qué recursos de información y apoyo puedo ofrecer a una mujer víctima de VG.	4,25	8,9	4,65	<0,001	2,1
	(2,07)	(0,99)	(4,24-5,06)		
7. Conozco las consecuencias de la VG para la salud de la mujer.	4,47	9,29	4,82	<0,001	2,12
	(2,04)	(0,84)	(4,40-5,24)		
8. Conozco la entrevista a realizar a una a posible víctima de VG.	4,22	8,89	4,54	<0,001	1,89
	(2,28)	(1,06)	(4,12-4,96)		
*Habilidad*					
1. Soy capaz cumplimentar partes de lesiones por VG	4,34	8,31	3,97	<0,001	1,82
	(1,36)	(1,36)	(3,57-4,38)		
2. Soy capaz de realizar preguntas de cribado de VG al atender a una mujer.	4,39	8,82	4,43	<0,001	1,91
	(2,25)	(1,00)	(4,00-4,85)		
3. Cuando he terminado de atender a la víctima de VG, soy capaz de ofrecer los recursos disponibles.	4,27	8,06	3,79	<0,001	1,66
	(2,15)	(1,09)	(3,37-4,21)		
*Actitud*					
1. Intento que otro compañero/a realice la atención a la víctima de VG.	8,43	8,63	0,2	0,003	0,28
	(1,13)	(0,99)	(0,07-0,33)		
2. En el último mes, he hablado con mis compañeros del tema de la VG desde el punto de vista de atención sanitaria.	6,77	7,52	0,75	<0,001	0,62
	(1,43)	(0,81)	(0,52-0,97)		
3. Me cuesta hablar con compañeros/as sobre el tema de la VG.	8,55	8,76	0,21	0,018	0,22
	(1,06)	(1,11)	(0,04-0,38)		
4. Creo que el abordaje de la VG debe ser tratado de forma interdisciplinar.	8,82	8,79	-0,03	0,657	-
	(1,16)	(1,12)	(-0,14-0,09)		
5. Me siento incomodo/a cuando estoy atendiendo a una posible víctima de VG.	8,38	8,7	0,31	<0,001	0,57
	(0,9)	(0,81)	(0,21-0,42)		
6. Me causa ansiedad saber que la mujer sufre maltrato pero que no quiere denunciar.	7,98	8,13	0,15	0,001	0,31
	(0,99)	(0,87)	(0,06-0,24)		

M: media; DE: desviación estándar; IC: intervalo de confianza; d: tamaño de efecto de Cohen; VG: violencia de género.

Las personas participantes puntuaron alto en las preguntas sobre la consolidación del aprendizaje (puntuaciones ≥9 sobre 10), sobre la aplicabilidad al puesto de trabajo (puntuaciones ≥8 sobre 10) y sobre si los conocimientos adquiridos fueron suficientes para aplicarlos al puesto de trabajo (Tabla 4); lógicamente, la pregunta *con lo aprendido en la actividad formativa he necesitado ampliar/profundizar en los contenidos para su aplicación práctica* obtuvo una puntuación media baja (2,16).

**Tabla 4:** Puntuaciones (media y desviación estándar) obtenidas en las preguntas sobre consolidación del aprendizaje, la aplicabilidad al puesto de trabajo y la de necesidad de formación adicional

*Consolidación del aprendizaje*	
*La acción formativa de abordaje de la violencia de género con metodologías activas de aprendizaje, me ha permitido:*	
Conocer los aspectos claves de la materia en cuestión.	9,19 (0,89)
Descubrir herramientas y técnicas útiles para mi trabajo.	9,16 (1,00)
Adquirir/reforzar mis competencias profesionales.	9,28 (0,90)
*Aplicabilidad al puesto de trabajo*	
*Sobre la acción formativa de abordaje de la violencia de género con metodologías activas de aprendizaje*	
De la formación recibida he aplicado a mi puesto de trabajo aspectos teóricos.	8,73 (1,09)
De la formación recibida he aplicado a mi puesto de trabajo aspectos prácticos.	8,65 (1,03)
La formación recibida la he aplicado en mi puesto de trabajo de forma habitual.	8,22 (1,32)
*Necesidad de formación adicional*	
Con lo aprendido en la actividad formativa he tenido suficiente para aplicarlo al puesto de trabajo	8,50 (1,11)
Con lo aprendido en la actividad formativa he necesitado ampliar/profundizar en los contenidos para su aplicación práctica	2,16 (0,88)

## DISCUSIÓN

El objetivo de este estudio fue evaluar el impacto de una formación basada en metodologías activas, *online* y, con la reflexión como punto de partida, adquirir una mejora en las competencias relacionadas con la gestión de los casos de violencia de género en los servicios de urgencias. Este trabajo contribuye a aumentar el conocimiento sobre la mejor manera de formar a los profesionales en este aspecto.

Numerosos estudios subrayan la necesidad de gestionar eficazmente los casos de violencia de género en los servicios de urgencias^26^^-^^29^. La alta prevalencia de situaciones de violencia de género en estos contextos clínicos^3^^,^^39^ demanda una intervención adecuada y una atención inmediata, haciendo crucial que los profesionales de la salud estén bien preparados para identificar y abordar estos casos de manera efectiva. La muestra de 115 profesionales sanitarios (compuesta mayoritariamente por mujeres enfermeras y médicas) refleja la composición típica de este ámbito laboral y destaca la importancia de focalizar la formación en estos grupos.

Los datos obtenidos demuestran que el programa de formación en violencia de género, que incluye reflexión guiada sobre vídeos didácticos y vídeos-problema dramatizados, es altamente efectivo. Se observó una mejora significativa en la evaluación autopercibida de las personas participantes en las dimensiones de *conocimiento* y *habilidad*, con tamaños del efecto moderados. Esto indica que las participantes consideran que adquirieron nuevos conocimientos y habilidades sobre el abordaje de las mujeres que sufren violencia de género. La dimensión de *actitud* también mostró mejoras significativas, aunque con un tamaño del efecto más pequeño, lo que sugiere que, si bien las actitudes hacia la violencia de género eran ya positivas al presentarse de forma voluntaria a la acción formativa, el programa logró fortalecer aún más esta perspectiva. Estudios previos^32^^,^^33^^,^^40^^,^^41^ en estudiantes de enfermería y medicina han avalado esta metodología que ahora aplicamos en profesionales de urgencias y emergencias.

La elevada puntuación de las personas participantes en las preguntas sobre la consolidación del aprendizaje y la aplicabilidad al puesto de trabajo refleja la eficacia del programa. Las puntuaciones superiores a 9 sobre 10 en la consolidación del aprendizaje y superiores a 8 sobre 10 en la aplicabilidad al puesto de trabajo indican que los contenidos del curso fueron percibidos como relevantes y útiles para su práctica diaria. La baja puntuación en la necesidad de formación adicional^12^^,^^16^ sugiere que el personal formado se sintió suficientemente capacitado con el contenido ofrecido, lo que destaca la efectividad del programa a pesar de ser breve y *online*.

Estos hallazgos son particularmente relevantes en el contexto actual, donde los recursos para la formación continua a menudo son limitados, o el personal no dispone del suficiente tiempo para formarse^42^. Existe una limitación curricular evidente en las profesiones sanitarias respecto a la violencia de género, lo que podría llegar a influir en la atención en los contextos clínicos reales^43^, siendo necesario el establecimiento de programas de formación específicos como este que presentamos. La capacidad de ofrecer un curso breve, online y con recursos no muy amplios, pero que aun así logre resultados significativos de percepción de aprendizaje, subraya la viabilidad y efectividad de este enfoque para que pueda ser replicado y adaptado a otros contextos y necesidades, ampliando así el impacto positivo en la calidad de la atención en casos de violencia de género.

La implementación de programas de formación que utilicen recursos didácticos innovadores como la inclusión de vídeos-problema dramatizados^33^^,^^40^ puede mejorar significativamente los conocimientos, habilidades y actitudes de profesionales de la salud, asegurando una mejor atención a las víctimas de violencia de género y contribuyendo a una gestión más eficaz de estos casos en el ámbito sanitario.

Se puede decir que el punto fuerte de este estudio ha sido el uso del diálogo (aprendizaje dialógico) para provocar la reflexión crítica grupal como herramienta fundamental. El potencial de este método ha sido ampliamente descrito^44^^-^^46^, aunque no suficientemente investigado en su aplicación clínica. El uso de la simulación en la formación ha sido uno de los pilares del aprendizaje desde hace décadas y actualmente se está configurando como uno de los anclajes más importantes del entrenamiento de los profesionales^47^. Si bien este estudio no contempla la dimensión experiencial de la simulación, sí que implica a las participantes en la reflexión guiada (*debriefing*)^48^ y en el aprendizaje observacional o vicario^49^. Creemos que los buenos resultados de las mediciones del instrumento tras la formación recibida son fruto del método pedagógico utilizado.

El presente estudio tiene algunas limitaciones. El diseño cuasiexperimental sin grupo control limita la atribución de los cambios exclusivamente a la intervención. Se realizó un muestreo intencional, no estadístico, lo cual limita la validez del estudio. La muestra de 115 participantes puede no ser generalizable a otros contextos, lo que limita su validez externa. La participación voluntaria pudo haber introducido un sesgo de autoselección. Los efectos a largo plazo de la formación no fueron evaluados, y la dependencia de autoinformes puede haber introducido sesgos de percepción. Además, aunque se utilizaron vídeos-problema dramatizados, no se evaluaron otras modalidades de formación alternativas como simulaciones clínicas con pacientes estandarizados, donde el discente no solo demuestra sus conocimientos, sino también su capacidad de abordaje con pacientes simulados. En estos contextos, las competencias se desarrollan, observan y reciben retroalimentación. Sin embargo, al utilizar vídeos problema inacabados en este estudio, el estudiante solo expresa su percepción sobre lo que sabe o conoce, sin llegar a demostrarlo en un entorno clínico real o simulado. Otra limitación del presente estudio fue el uso de cuestionarios autoadministrados como herramienta de evaluación, que no permite una evaluación objetiva de la adquisición de conocimientos y habilidades antes y después de la actividad formativa. Aunque son métodos válidos, presentan ciertos sesgos de medición, como el sesgo de deseabilidad social, donde los participantes pueden responder de manera que consideren más aceptable, y el sesgo de interpretación, ya que la comprensión de las preguntas puede variar entre los encuestados. Aunque durante la medición se intentó controlar estos sesgos, pueden haber influido en la precisión de las respuestas y, por tanto, en la validez de los resultados. Es importante considerar estas limitaciones al interpretar los hallazgos, y estudios futuros podrían complementarse con métodos observacionales para una evaluación más completa. Finalmente, no se incluyó una evaluación directa del impacto clínico en la práctica diaria ni en los resultados de salud de los pacientes, lo que abre la puerta a seguir investigando con más profundidad en estudios prospectivos.

En conclusión, este estudio ha demostrado la eficacia de una formación *online* basada en metodologías activas y reflexión para mejorar las competencias en la gestión de casos de violencia de género en urgencias y emergencias, sin diferencias por sexo. Los resultados revelan una mejora significativa en conocimientos, habilidades y actitudes de profesionales sanitarios. La alta puntuación en la consolidación del aprendizaje y aplicabilidad al trabajo reafirma la utilidad del curso. Estos hallazgos subrayan la viabilidad de ofrecer cursos breves y *online* con recursos innovadores como vídeos-problema dramatizados para capacitar a profesionales, incluso con recursos limitados. La formación continua y específica en violencia de género es crucial para garantizar una atención eficaz en urgencias y emergencias, mejorando la calidad del cuidado y la gestión de estos casos en el ámbito sanitario.

## Data Availability

Se encuentran disponibles bajo petición al autor de correspondencia.

## ANEXO I

**Table:** Cuestionario de conocimientos, habilidades y actitudes sobre violencia de género (CCHA-VioGen)^30^

Ítem	Dimensión
	*Conocimiento*
1	Conozco el protocolo sanitario de VG.
2	Conozco cómo actuar al encontrarme en mi servicio con una posible víctima de VG.
3	Conozco las preguntas de cribado para identificar a una posible víctima de VG.
4	Conozco los riesgos en la mujer que es víctima de VG.
5	Conozco cómo cumplimentar y qué hacer con el parte de lesiones ante una víctima de VG.
6	Conozco qué recursos de información y apoyo puedo ofrecer a una mujer víctima de VG.
7	Conozco las consecuencias de la VG para la salud de la mujer.
8	Conozco la entrevista a realizar a una a posible víctima de VG.
	*Habilidad*
9	Soy capaz cumplimentar partes de lesiones por VG.
10	Soy capaz de realizar preguntas de cribado de VG al atender a una mujer.
11	Cuando he terminado de atender a la víctima de VG, soy capaz de ofrecer los recursos disponibles.
	*Actitud*
12	Intento que otro colega realice la atención a la víctima de VG.
13	En el último mes, he hablado con mis colegas del tema de la VG desde el punto de vista de atención sanitaria.
14	Me cuesta hablar con colegas sobre el tema de la VG.
15	Creo que el abordaje de la VG debe ser tratado de forma interdisciplinar.
16	Siento incomodidad cuando estoy atendiendo a una posible víctima de VG.
17	Me causa ansiedad saber que la mujer sufre maltrato pero que no quiere denunciar.

VG: violencia de género

La dimensión *conocimiento* evalúa, mediante ocho ítems, el conocimiento sobre el protocolo de salud de la violencia de género, sobre las posibles acciones cuando se trata de una víctima de violencia de género, sobre las preguntas de selección para identificar a una posible víctima de violencia de género, los riesgos de una mujer que es víctima de violencia de género, cómo completar y qué hacer con el informe de lesión cuando se trata de una víctima de violencia de género, qué información y apoyo podría ofrecerse a una mujer víctima de violencia de género, las consecuencias de la violencia de género en la salud de la mujer, y una entrevista que se llevará a cabo con una posible víctima de violencia de género.

La dimensión *habilidad* evalúa, mediante tres ítems, aspectos de las personas participantes como su capacidad para completar el informe de lesiones de las mujeres víctimas de VG, hacer las preguntas de cribado de VG y ofrecer los recursos disponibles.

La dimensión *actitud* evalúa, mediante seis ítems, la posición del personal médico y enfermero participante sobre la atención de la violencia de género, el abordaje con colegas desde el punto de vista de la atención a la salud, si sentían incomodidad y si experimentaban ansiedad al atender a una posible víctima de violencia de género que no quería denunciar al agresor.

## ANEXO II

**Preguntas adicionales elaboradas *ad hoc* para evaluar la mejora en las herramientas para abordar la violencia de género en los servicios de urgencias/emergencias después de la acción formativa**

**Consolidación del aprendizaje**

La acción formativa de abordaje de la violencia de género con metodologías activas de aprendizaje, me ha permitido:

- Conocer los aspectos claves de la materia en cuestión.
- Descubrir herramientas y técnicas útiles para mi trabajo.
- Adquirir/reforzar mis competencias profesionales.

**Aplicabilidad al puesto de trabajo**

Sobre la acción formativa de abordaje de la violencia de género con metodologías activas de aprendizaje:

- De la formación recibida he aplicado a mi puesto de trabajo aspectos teóricos.
- De la formación recibida he aplicado a mi puesto de trabajo aspectos prácticos.
- La formación recibida la he aplicado en mi puesto de trabajo de forma habitual.

**Necesidad de formación adicional**

- Con lo aprendido en la actividad formativa he tenido suficiente para aplicarlo al puesto de trabajo.
- Con lo aprendido en la actividad formativa he necesitado ampliar/profundizar en los contenidos para su aplicación práctica.
